# Reactive oxygen species within the vaginal space: An additional promoter of cervical intraepithelial neoplasia and uterine cervical cancer development?

**DOI:** 10.1515/med-2023-0826

**Published:** 2023-10-21

**Authors:** Albert Despot, Rajko Fureš, Ana-Marija Despot, Mislav Mikuš, Gordan Zlopaša, Antonio D’Amato, Vito Chiantera, Pietro Serra, Andrea Etrusco, Antonio Simone Laganà

**Affiliations:** School of Medicine, University of Zagreb, 10000 Zagreb, Croatia; Department of Obstetrics and Gynecology, General Hospital Zabok, 49210 Zabok, Croatia; Faculty of Food Technology and Biotechnology, University of Zagreb, 1000 Zagreb, Croatia; Department of Obstetrics and Gynecology, Clinical Hospital Center Zagreb, 1000 Zagreb, Croatia; Unit of Obstetrics and Gynecology, Department of Biomedical and Human Oncological Science, University of Bari, 70100 Bari, Italy; Department of Health Promotion, Mother and Child Care, Internal Medicine and Medical Specialties (PROMISE), University of Palermo, 90127 Palermo, Italy; Unit of Gynecologic Oncology, National Cancer Institute – IRCCS – Fondazione “G. Pascale”, 80131 Naples, Italy; Unit of Obstetrics and Gynecology, “Paolo Giaccone” Hospital, 90127 Palermo, Italy

**Keywords:** cervical cancer, cervical intraepithelial neoplasia, oxygen reduction, oxidative stress, vaginal microbiota

## Abstract

Uterine cervical intraepithelial abnormalities and cancer development may also depend upon biological problems that arise as a result of complex molecular disturbances within the vaginal space, in addition to the widely known causative effect of human papillomavirus (HPV) infection. Chronic oxidative stress is a consequence of oxygen reduction in the vaginal space. Reactive oxygen species (ROS) and free radicals are yet unrecognizable causative agents and are probably very important factors for cervical intraepithelial neoplasia (CIN) and cancer development. The intermediate compounds of oxygen reduction on these metabolic pathways are superoxide anion ( \[{\text{O}}_{2}^{ \textdotaccent -}]\] ), hydrogen peroxide (H_2_O_2_), hydroxide ions (OH^−^), and hydroxyl radical (HO˙). Considering these points, the aim of this work was to summarize how these compounds can damage all molecules, including DNA, of vulnerable metaplastic cervical epithelium. Finally, in some women with a poor immune defense system, ROS alone or accompanied by a high-risk HPV type may promote all levels of CIN and cancer development.

## 1 Introduction

Cervical cancer is a global health problem. According to the latest data, 604,127 new cases and 341,831 deaths from cervical cancer were reported worldwide in 2020 [1]. Compared to reports from 2018, the frequency increased by 35,000 new cases and mortality by 30,000 women. All statistical indicators have shown an upward trend on graphical curves. In Europe, approximately 61,000 women develop uterine cervical cancer annually, resulting in approximately 26,000 deaths [2].

Despite classical diagnostic and therapeutic protocols (PAP smear, human papillomavirus [HPV] typing, colposcopy, biopsy, radical surgery, chemotherapy, and radiotherapy), cervical cancer still has a high level of incidence and mortality worldwide [2].

To date, recent investigations of vaginal *Lactobacillus* species and their hydrogen peroxide (H_2_O_2_) production have shed new light on this topic [3]. The H_2_O_2_ produced by *Lactobacillus* can catalyze and further produce hypochlorite [4]. This is a process that can prevent HPV from invading cervical epithelial cells and prevent cervical lesions [5]. Nevertheless, *Lactobaccilus* species are anaerobic bacteria without a described biological system for hydrogen peroxide production.

In addition, hydrogen peroxide (H_2_O_2_) production is only one-fourth of the well-known *univalent pathway* of ground-state oxygen reduction to water (H_2_O) through intermediate compounds such superoxide ( \[{\text{O}}_{2}^{ \textdotaccent -}]\] ), hydrogen peroxide (H_2_O_2_), and the hydroxyl radical (OH) plus hydroxide ions (OH^−^) inside the vaginal space.

Third, the detection of hydrogen peroxide (H_2_O_2_) in the vagina is proof that this space can be influenced by reactive oxygen species (ROS) and free radicals.

Overall, current etiological and carcinogenesis explanations from molecular biology standpoints need further investigation.

Considering these points, we aimed to summarize the available pieces of evidence, hypothesizing whether the reduction of oxygen to ROS within the vaginal space may be a further promoter of cervical intraepithelial neoplasia (CIN) and/or the development of cervical cancer and what mechanisms underlie this phenomenon.

In particular, the hypothesis is discussed in the next sections, starting with the current knowledge on the correlation between HPV infection and cervical cancer, then moving on to assess the mechanisms of transformation from CIN to low- or high-grade squamous lesion (LSIL/HSIL), and finally to examine the potential effect that vaginal lactic acid may in some way have on the development of cervical cancer.

## 2 HPV and cervical cancer

Recent studies of the uterine cervical cancer incidence in Sweden and Denmark have added further insight into the classic model of its carcinogenesis. A Swedish study found that the increase in cervical cancer incidence was high among women repeatedly screened with normal results and not among unscreened women [6]. The reason has been found to be insufficient cytological diagnostic activities inside county laboratory mismanagement. On the other hand, after organized HPV vaccination at a youth clinic in Stockholm, nonvaccine high-risk HPV types remained at elevated levels [7].

In neighboring Denmark, HPV vaccination at the age of 23–24 years has produced a 95% decrease in comparison with the prevalence inside the same population of women before the vaccination era. Despite this impressive reduction, 35% of women after vaccination have remained HPV-positive [8], and high-grade cervical dysplasia may occur even in HPV-negative women [9].

Recently, published studies of HPV infection rates in postmenopausal women showed virus presence rates of 4.1% in Sweden [10] and 4.3% in Denmark [11]. The results of studies in Sweden and Denmark were very similar. It is paradoxical that HPV infection incidence in postmenopausal women has decreased, but the uterine cervical cancer rate has unexpectedly increased at the same time within the same population. A meta-analysis of real worldwide studies revealed that 12.7% of squamous cell carcinomas and 15–38% of cervical adenocarcinomas are HPV-negative [12–14].

In this scenario, other biological elements may be considered adjuvants for the initiation of cervical carcinogenesis.

## 3 Transformation from CIN to LSIL/HSIL

Over the last 50 years, our increased understanding of uterine cervical cancer pathogenesis has evolved [15,16]. HPV is a unique etiological factor for CIN as well as cancer development [17,18]. The strong correlation between woman’s age and CIN has been shown in almost all investigations and meta-analyses [19]. Investigations have revealed that the usual time for CIN events is from 20 to 30 years in women’s lives. In that period of life, sexual activity is more common than it is sooner or later.

*Bluestein’s Pathology of Female Genital Tract* (sixth edition) reports a meta-analysis of clinical follow-up studies of biopsy-confirmed CIN published in the mid-1990s [20,21]. According to this meta-analysis, in the absence of therapy, “the higher grade of lesion is more likely to persist and less likely to regress.” In addition, 22% of CIN II cases progress to carcinoma *in situ* (CIS), but CIN III has an unexpected progression rate of 12% to CIS.

Another recent meta-analysis, based on a large population study with 783 patients using histological data, found that the regression rates are notably higher in young women with CIN and showed that the effect is independent of CIN grade or the presence of HPV high-risk infection. Overall, this article highlighted that the progression rates for CIN I, CIN II, or CIN III were uncommon [22].

Recently, more and more evidence has accumulated suggesting that during progression to high-grade intraepithelial neoplasia and cervical cancer, nuclear factor kappaB (NF-kB) is constitutively reactivated [23]. The NF-kB family is composed of transcription factors that play a complex and essential role in the regulation of immune responses and inflammation. In particular, NF-kB can stimulate transcription of proliferation-regulating genes (e.g., cyclin D1 and c-myc) involved in metastasis, VEGF-dependent angiogenesis, and cell immortality through telomerase [24]. It has also attracted considerable interest because it has been implicated in human cancer initiation, progression, and treatment resistance [24,25]. NF-kB activation, triggered by HPV infection, plays an important role in the host innate and adaptive immune response. The virus induces a down regulation of NF-kB to eliminate the inhibitory activity for its replication triggered by the immune system, resulting in a persistent state of HPV infection. During progression to high-grade intraepithelial neoplasia and cervical cancer, NF-kB is constitutively reactivated. NF-kB activation can also induce the expression of activation-induced cytidine deaminase and APOBEC proteins, providing a mechanistic link between the NF-kB pathway and the mutagenic features of cervical cancer [23].

Mitochondrial dysfunction is also known to be involved in tumorigenesis [26]. In recent years, the mitochondrial ND1 (mtND1) gene has received considerable interest from the scientific community. Recently identified mutations in the mtND1 gene have been shown to disrupt normal complex I activity and affect oxidative phosphorylation, thus leading to increased ROS production. Based on these elements, a study was conducted to determine the alterations in the mtND1 gene and evaluate their association with the development of precancerous lesions and cervical cancer. Warowicka et al. [27] showed that mutations in the mtND1 gene were detected in patients with precancerous stage and cervical cancer. The lowest mRNA expression of ND1 was detected in cervical cancer cases and in all samples in which mtND1 mutations were identified. The analysis also revealed that the level of ROS production was higher in cervical cancer tissues and in all cases characterized by mtND1 mutations.

From the clinical point of view, vaginal space is rich in all chemical resources for oxygen activation and its transformation through reduction to water. The complete reduction of oxygen to water requires four electrons and four protons to be added to oxygen in a stepwise chemical path, better known as a univalent pathway. It requires the production of intermediate compounds such as superoxide (O_2_^•–^), hydrogen peroxide (H_2_O_2_), the hydroxyl radical (˙OH), and hydroxide ions (OH^−^). These compounds are the main part of ROS. These intermediate compounds can damage all cell structures, including DNA, and may play a significant role in cervical cancer pathogenesis, as described in the next section.

## 4 Potential vaginal lactic acid role for cervical cancer pathogenesis

The lactic acid inside the vaginal space has two different metabolic pathways. The first is a well-known physiological path built in biological collaboration between *Lactobacillus* species and women. The woman as the host provides glycogen to anaerobe bacteria. *Lactobacillus* species use it as a life nutrient for their own growth and in turn convert glycogen from exfoliated superficial vaginal and uterine ectocervix cells to lactic acid.

Hydronium ion [H_3_O^+^] or proton [H^+^] concentrations are indicators of the health status in the lower part of the woman’s reproductive tract. During fertile age, the average pH of the cervico-vaginal fluid samples is 3.5, with a range of 2.8–4.2 [28].

*Lactobacillus* exhibits a mutualistic relationship with the woman’s body, as it protects the host against potential invasions by pathogens, and in turn, the host provides a source of nutrients to support bacterial growth [29,30].

*Lactobacillus* species convert glycogen from exfoliated superficial vaginal and exocervix cells to lactic acid. The cervicovaginal partial pressure of O_2_ is in the range of 4–14 mmHg (0.5–1.8%), with only transient increase during vaginal intercourse and some gynecological diagnostic activities [28]. In the air environment, the partial pressure of oxygen (O_2_) is 160 mmHg or 21%. Lactobacilli use exfoliated cells rich in glucose as a metabolic ingredient and energetic supply. During reproductive age, this process continues because the vaginal epithelium, under ovarian estrogen influence, is regenerated every 4–5 days. At the end of this physiological pathway, the vaginal environment is supplied with a high concentration of lactic acid (CH_3_CHOHCOOH). After lactic acid dissociation, the vaginal space has the same concentrations of hydronium ions [H_3_O^+^] and lactate [CH_3_CH(OH)COO^−^].

The lactic acid needs water for dissociation: CH_3_CHOHCOOH + H_2_O = [CH_3_CHOHCOO^−^] + [H_3_O^+^].

High concentration of [H_3_O^+^] in vaginal space creates physiological pH level at 3.5. In such physiological condition, vaginal ecosystem has effective defense network against external microorganisms.

During reproductive years the vaginal pH should be 3.5 and the pOH 10.5, respectively, because the sum of pH and pOH in all solutions has the same numerical indicator of 14. In the first step, from the *Henderson–Hasselbalch* equation, it is necessary to determine the concentration of hydronium ions [H_3_O^+^] and hydroxide ions [OH^−^] in aqueous solution at physiological pH level:

pH = 3.5

pOH = 10.5

[H_3_O^+^] = 10^−3.5^ = 3.1623 × 10^−4^ mol/L

N[H_3_O^+^] = Na·m = 6.022141 × 10^23^ × 3.1623 × 10^−4^ = 1.90436 × 10^20^

[OH^−^] = 10^−10.5^ = 3.16227 × 10^−11^ mol/L

N[OH^−^] = Na·m = 6.022141 × 10^23^ × 3.16227 × 10^−11^ = 1.90436 × 10^13^

Based on the *Henderson–Hasselbalch* equation and biochemistry relationships, the concentration of hydronium ions [H_3_O^+^] or protons [H^+^] is 1.9 × 10^7^ or 19 million times higher than the concentration of hydroxide ions [OH^−^] at physiological vaginal pH value of 3.5. The lactic acid and lactate concentration is calculated by the same equation as follows:

pH = 3.5

pKa (CH_3_CH(OH)COOH) = 3.86

Ka = 10^−3.86^ = 1.3804 × 10^−4^ mol/L

(CH_3_CH(OH)COOH) = 7.2444 × 10^−4^ mol/L (initial concentration)

CH_3_CH(OH)COOH + H_2_O = CH_3_CH(OH)COO^−^ + H_3_O^+^

Ka = [H_3_O^+^] [CH_3_CH(OH)COO^−^]/(CH_3_CH(OH)COOH)

[H_3_O^+^] = [CH_3_CH(OH)COO^−^] = 10^−pH^ = 10^−3.5^ = 3.16227 × 10^−4^ mol/L

Further analysis follows calculation of lactate CH_3_CH(OH)COO^−^ and lactic acid CH_3_CH(OH)COOH concentrations.

Na(CH_3_CH(OH)COO^−^) = Na·m = 6.022141 × 10^23^ × 3.1623 × 10^−4^ = 1.90436 × 10^20^

Na(CH_3_CH(OH)COOH) = Na·m = 7.2444 × 10^−4^ × 6.022141 × 10^23^ = 4.36268 × 10^20^

From a biological point of view, the architecture of vaginal physiology is quite relativistic. The vaginal space is hypoxic. The oxygen molecules in the triplet ground state have no toxic effect on their environment. For toxic transformations, the oxygen needs energy activation. The lactic acid dissociation to hydronium ions [H_3_O^+^] and lactate [CH_3_CH(OH)COO^−^] is the energetic fuel for potential toxic oxygen turn over. The oxygen needs four protons [H^+^] and four electrons [e^−^] for its reduction to water. The protons have origin from hydronium ions [H_3_O^+^], but the electrons have origin from lactate [CH_3_CH(OH)COO^−^].

A high lactate concentration is the source for extensive electron donation in the *Lewis concept* of acids and bases. Reduction is the gain of hydrogen, gain of electrons, and loss of oxygen. Reduction of ground state triplet oxygen is the basic process of oxidative stress and ROS production. ROS are classically defined as partially reduced metabolites of oxygen that possess strong oxidizing capabilities. They are deleterious to cells at high concentrations, but at low concentrations, they serve as complex signaling functions at the physiological level [31].

ROS oxidizes protein and lipid cellular constituents and may also damage the DNA. At low physiological concentrations, ROS functions as signaling molecule and regulate cell growth, adhesion of cells toward other cells, differentiation, senescence, and apoptosis [32,33].

The intermediate compounds of oxygen reduction on the univalent pathway are superoxide ( \[{\text{O}}_{2}^{ \textdotaccent -}]\] ), hydrogen peroxide (H_2_O_2_), the hydroxyl radical (˙OH), and hydroxide ions (OH^−^) [34].

The additional problem of this complexity is the life expectancy of free radicals. It depends on two main factors. The former is more chemical than biological in nature. A measure of life expectancy in chemistry is the half-life. In general, free radicals are short-lived, especially in biological milieu. The half-life of free radicals, including the vaginal cornerstone compound superoxide anion ( \[{\text{O}}_{2}^{ \textdotaccent -}]\] ), is an important factor in determining and assessing the biological activity of radical species.

In biological systems, the half-life of superoxide is estimated to be in the range of 10^−6^–10^−5^ s. Sometimes something that seems short-lived is unusually long-lived, especially if we add new possibilities for ROS development from superoxide anion ground level, in chemistry better known as *Haber–Weiss and Fenton reactions*.

## 5 Oxidative stress, CIN, and cancer

Molecular dioxygen (O_2_) is an essential element of aerobic life, yet incomplete reduction or excitation of O_2_ during aerobic metabolism generates diverse oxygen-containing free radicals and related reactive species, commonly known as ROS.

On the one hand, these compounds are a serious threat to aerobic organisms by inducing oxidative damage to cellular constituents. On the other hand, these reactive species, when their generation is under homeostatic control, also play important physiological roles through participation in redox signaling and as an important part of immunity [33].

Oxidative stress conditions can be caused by either increased ROS formation, decreased activity of antioxidants, or both in a biological system. Oxidative stress accompanied by chronic inflammation can produce all kinds of cell damage, including cancer initiation/development.

Extensive research during the last two decades has revealed the mechanism by which continued oxidative stress can lead to chronic inflammation, which in turn could mediate most chronic diseases, including cancer, diabetes, cardiovascular, neurological, and pulmonary diseases [35]. From this perspective, extensive production of ROS inside the vaginal space throughout life may be a trigger for the development of diverse cervical intraepithelial abnormalities.

The electronic structure for ground state oxygen, which is essential for the life of all aerobic organisms, makes it potentially dangerous for those organisms. The atmospheric oxygen in its triplet ground state is biradical (i.e., it has two unpaired electrons). This feature makes oxygen paramagnetic, but the two unpaired electrons in oxygen occupy a separate orbital. These two unpaired electrons have the same spin or spin state. According to *Pauli*’s exclusion principle such paired electrons with same parallel rotation produce the spin restriction. This means that the triplet ground state of oxygen makes it very unlikely to participate in reactions with organic molecules unless it is “activated.” In different microenvironment, the activation of oxygen may occur by two different mechanisms:

1. Absorption of sufficient energy to reverse the spin on one of the unpaired electrons.
2. Through mono- or univalent reduction.

The vaginal space is rich with all chemical resources for oxygen activation and its transformation through reduction to water. The complete reduction of oxygen to water requires four electrons and four protons to be added to oxygen in a stepwise chemical fashion producing intermediate compounds as follows:

1. O_2_ (oxygen molecule) + e^−^ (electron) = \[{\text{O}}_{2}^{ \textdotaccent -}]\] (superoxide ion)
2.
\[{\text{O}}_{2}^{ \textdotaccent -}]\] (superoxide ion) + 2H^+^ (protons) + e^−^ = H_2_O_2_ (hydrogen peroxide)
3. H_2_O_2_ + e^−^ = OH* (hydroxyl radical) + OH^−^ (hydroxide ion)
4. OH* + OH^−^ + e^−^ + 2H + = 2H_2_O (water)

For complete reduction of an oxygen molecule to two molecules of water requires four electrons and four protons to be added to the oxygen molecule. The biological pathway shown through four stepwise reactions is also known and as “the univalent pathway.” The path described is not the end of ROS production. Since 1933 when *Linus Pauling* proposed superoxide existence based on the theory of quantum mechanics, this has gradually taken the center stage in the research field of free radical biology and medicine. Indeed, superoxide is considered as the primary ROS that gives rise to secondary ROS in biological system. Due to its low reductional potential, superoxide often acts as reducing agent, rather than oxidizing species. Superoxide is produced from one electron reduction of molecular dioxygen (oxygen for simplicity) [36]: O_2_ + e^−^ = \[{\text{O}}_{2}^{ \textdotaccent -}]\] . In biological systems, superoxide is generated from various metabolic processes. Molecular oxygen (O_2_) is in fact biradical because it contains two unpaired electrons. The same spin direction of two unpaired electrons in O_2_ causes spin restriction, making O_2_ less reactive. On the other hand, superoxide ( \[{\text{O}}_{2}^{ \textdotaccent -}]\] ) contains one unpaired electron and has one more electron other than O_2_, so it means that superoxide is negatively charged. In such environmental circumstances, a superoxide anion radical can donate one electron to molecule of hydrogen peroxide leading to the formation of one molecule of molecular oxygen, a hydroxyl radical, and a hydroxide ion as follows: \[{\text{O}}_{2}^{ \textdotaccent -}]\] + H_2_O_2_ = O_2_ + OH* + OH^−^.

This is also known as *Heber–Weiss* reaction or *Heber–Weiss cycle* [37]. In the absence of iron ion (Fe^3+^) the above-mentioned reaction proceeds very slowly. However, the presence of iron ion (Fe^3+^) markedly accelerates the reaction to produced hydroxyl radical (OH*), the most potent ROS capable of damaging the entire spectrum of biomolecules. The presence of iron ions markedly accelerates the reaction to produce hydroxyl radical. The iron-ion catalyzed Heber–Weiss reaction can be written in two sequential sub-reactions:

1.
\[{\text{O}}_{2}^{ \textdotaccent -}]\] + Fe^3+^ = O_2_ + Fe^2+^
2. Fe^2+^ + H_2_O_2_ = Fe^3+^ + OH* + OH^−^

The second reaction is commonly referred to as the *Fenton reaction*. It represents the oxidation of organic substrates by Fe^2+^ and H_2_O_2_. In 1894, Henry Fenton first observed the oxidation of tartaric acid by H_2_O_2_ in the presence of Fe^2+^, indicating that potent oxidant(s) are formed by the reaction between H_2_O_2_ and Fe^2+^, which are also known as the *Fenton reagent*.

Later, in the 1930s, Fritz Haber and Joseph Weiss proposed the formation of hydroxyl radicals from the *Fenton reagent*, which is today known to be a major ultimate free radical species responsible for the oxidative damage of a wide range of biomolecules. In general, free radicals are short-lived in biological milieu.

The half-life of superoxide is an important factor in determining the biological activity of radical species. The half-life of superoxide is estimated to be in the range of 10^−6^–10^−5^ s. For these reasons, increasing concentrations of superoxide anions in vaginal space have time for hydroxyl radical production through *Haber–Weiss and Fenton reactions*.

In addition, any increase in exposure to iron ions, as it occurs during the menstrual period, supports oxidative stress, and markedly accelerates superoxide anion transformation to hydroxyl radicals (OH*), the most potent oxidizing ROS formed in biological systems. According to recent data, it is also known that ROS can induce DNA double-strand breaks in both host DNA and the viral circular episome; this could facilitate virus integration, promoting HPV carcinogenesis [38]. Therefore, in HPV-infected women, it might be beneficial to reduce additional ROS resources by improving the lifestyle. In this regard, it seems worth noting that modulation of the vaginal/cervical microbiome by exogenous administration of *Lactobacillus crispatus* has been associated with a high rate of HPV clearance. Indeed, recent studies have found a higher rate of clearance of PAP smear abnormalities in patients who took *Lactobacillus crispatus* M247 orally for a long time compared with the control group who had a simple follow-up [3]. Specifically, after a median follow-up of 12 months, a higher likelihood of resolution of HPV-related cytological abnormalities was found in patients on long-term oral probiotic treatment compared with the group who only did follow-up (60.5 vs 41.3%) [3]. Complete HPV clearance was evidenced in only 9.3% of patients on follow-up as compared to 15.3% of patients taking long-term oral *Lactobacillus crispatus* M247[3].

## 6 Consequences of the hypothesis and future research priorities

There are several different protocols for diagnostic approaches and treatments for CIN and cervical cancer worldwide.

The *Bethesda system* (TBS) was modified in 1991 and 2001 [39]. The last TBS classification subdivides atypical squamous cells into two subdivisions.

Atypical squamous cells of undetermined significance refer to samples in which the cytological changes are suggestive of LSIL, but atypical squamous cells cannot exclude HSIL [40].

The vaginal space, as the origin of ROS and free radicals, is not yet fully recognized as an adjuvant trigger for cervical intraepithelial abnormality development. Increasing oxygen concentrations accompanied by lactic acid dissociation are the source for the activation of the triplet ground state of molecular oxygen.

Oxygen activation in the vaginal space led to its reduction via a univalent pathway in a stepwise fashion, enriched with an additional four protons [H^+^] and an additional four electrons [e^−^] as well as superoxide ( \[{\text{O}}_{2}^{ \textdotaccent -}]\] ), hydrogen peroxide (H_2_O_2_), the hydroxyl radical (˙OH), and hydroxide ion (OH^−^) production. These intermediate compounds may influence the vulnerable metaplastic cervical epithelium.

From this perspective, it is possible that oxidative stress within the vaginal space may increase the risk that metaplastic cervical epithelium, caused by HPV infection, progresses to CIN and overt cervical cancer. Overall, this hypothesis needs to be evaluated by rigorous biochemical evaluation with future clinical trials. Future studies should therefore ascertain the actual applicability of the hypothesis to clarify its potential role in clinical practice. Should this be confirmed, the preventive focus of cervical cancer could extend from the microbiological and immunological realm to the biochemical field.

In addition, the possible confirmation of the hypothesis could open possible explanatory scenarios to understand how even in women in whom prevention of HPV infection, and thus screening for cervical cancer, is optimally performed, neoplasia can still develop.
